# Communicating radon risk via a smartphone app: a pilot intervention study

**DOI:** 10.1186/s12889-020-08677-7

**Published:** 2020-04-22

**Authors:** Soojung Kim, Michael S. Brewster, Gary G. Schwartz

**Affiliations:** 1 Department of Communication, University of North Dakota College of Arts & Sciences, Grand Forks, ND USA; 2 Triad Interactive Media, Greensboro, NC USA; 3 Department of Population Health, University of North Dakota School of Medicine & Health Sciences, 1301 N. Columbia Rd. Stop 9037, Grand Forks, ND 58202-9037 USA

**Keywords:** Radon, Smartphone, Lung cancer, Prevention, Risk communication

## Abstract

**Background:**

Residential radon is a major preventable cause of lung cancer. However, prevention requires radon testing and it has proven very challenging to motivate individuals to test their homes for hazards like radon that are invisible and whose health effects occur after a long latency following exposure. Novel approaches to radon communication are urgently needed.

**Methods:**

We created a novel radon-education app for smartphones and examined its effectiveness in increasing radon knowledge and radon testing. We studied radon knowledge and attitudes and behavior relevant to radon testing before and after app use.

**Results:**

Ninety-seven undergraduates installed the app on their smartphones and used it for a month. App use resulted in higher scores in the domains of radon knowledge (*p* < .001); self-efficacy (*p* < .001), and response efficacy (*p* < .001). Twenty-three participants (24%) used the app to obtain a free radon test kit. Self-efficacy (*p* < .05) and response efficacy (*p* < .01) were positive predictors of ordering a test kit. The test process completion rate (the fraction of participants who ordered test kits, used them to test their houses and sent the kits to the lab) was 9%.

**Conclusions:**

A smartphone app is a promising venue for communicating radon risk and for stimulating radon testing. Future interventions designed to increase actual test kit use are required to maximize the benefit of the app.

## Background

Radon gas is a form of ionizing radiation that results from the natural decay of radioactive elements thorium and uranium present in rocks and soils. It is the largest cause of lung cancer after smoking and causes more than 21,000 deaths per year in the U.S. [1]. The number of lung cancer deaths caused by radon far exceeds the number of deaths from more-publicized dangers, e.g., drunk driving (17,400) [2]. Indeed, the death toll from lung cancer likely underestimates radon’s force of mortality as radon also causes potentially fatal non-malignant lung disease, e.g., interstitial lung fibrosis [3] and may contribute to death from other cancers, e.g., chronic lymphocytic leukemia [4, 5] and malignant melanoma [6].

Deaths from radon ultimately derive from a failure to test homes for radon, which, in turn, reflects the public’s poor understanding of this hazard. A recent review of radon awareness in the U.S. indicated that most respondents do not know that radon causes lung cancer. Moreover, the majority of individuals younger than 30 years old do not even know what radon is [7].

Several studies have developed educational interventions concerning radon and a few have tested their effectiveness [8–12]. To the extent that interventions have been tested, most have performed poorly when measured against “real world” outcomes, such as increases in testing rates and home remediation. Thus, new approaches to radon communication are urgently needed [7, 13].

Considering that 81% of U. S adults in 2019 own a smartphone [14], communicating radon risks via a smartphone application (app) could be a novel approach to the problem of low radon awareness and testing. Smartphones and mobile applications have produced positive behavioral responses in diverse medical settings [15]. We therefore developed an app focused on radon awareness. This paper describes the radon app and presents results regarding the feasibility and effectiveness of the radon app intervention, focusing on its ability to engage users in using the app, learning about radon, and gauging their willingness to order a free radon test kit, use it, and return it to the lab for analysis.

### The radon app

Our app was designed to share existing content about radon and to distribute radon-related news reports as well as original informative content. The app allowed users to interact with content as well as request a free radon test kit. The technical work of designing the stand-alone mobile app was performed by Triad Interactive Media (Greensboro, NC). Briefly, Triad Interactive Media used a Progressive App framework in HTML5 and the open source Twitter API. Posts created by the app were cross-listed on Twitter, which allowed users to share conversations with a wide audience. The information hub of the app is its landing page, which posts radon-related content.

Once participants log in via their Twitter account, they can view recent radon news. The first menu, “In the News”, displays radon-related news curated from various sources (e.g., American Association of Radiation Scientists and Technologists, Environmental Protection Agency [EPA], and others). Participants also were exposed to additional original content that we provided about radon three to four times per week (“Team Posts”). This content was created by transforming existing content, such as the EPA brochure on radon information for homeowners [2], into a mobile-friendly format. Additionally, participants could access existing Tweets containing hashtags, e.g., #radon, #radonawareness, #radonmitigation, and #lungcancer. Participants could interact with any app content via Twitter’s user-engagement functions (Like, Comment, and Retweet). Participants also could perform tasks, such as filtering the app’s content, e.g., by hashtag, and could request a free radon test kit.

### Theoretical underpinnings of app feasibility and hypotheses

We hypothesized that the use of the radon app would increase individuals’ levels of radon knowledge and promote positive attitudes about radon testing. Attitudes are believed to be a strong predictor of behavioral changes [16]. Attitudes toward a behavior are defined as “an evaluation of performing a future behavior” [17] and the evaluation is expressed in terms of “favor or disfavor, good or bad, like or dislike” [18]. Improved radon knowledge and positive attitudes were expected to motivate individuals to order the test kit and to test their homes.

Similarly, we hypothesized that app use would increase the extent to which individuals evaluated radon exposure as a threat (i.e., threat appraisal) and radon testing as a way to cope with that threat (i.e., coping appraisal). Threat appraisal includes two concepts: perceived severity and perceived susceptibility. Perceived severity is the extent to which an individual perceives that a threat has negative consequences, whereas perceived susceptibility is the extent to which an individual believes that *he/she* personally is vulnerable to that threat. Perceptions of severity and susceptibility are expected to lead to adaptive responses such as ordering a radon test kit and testing the home [19, 20]. Second, coping appraisal includes response efficacy, which refers to an individual’s evaluation of the effectiveness of recommended behavior in terms of preventing a threat (e.g., ordering and testing behaviors). Another component of coping appraisal, self-efficacy, refers to an individual’s perceived capability to perform a recommended behavior. Thus, we hypothesized that the use of the  app would result in increased response efficacy and self-efficacy and promote individuals to perform the recommended responses, obtaining a test kit and using it [9, 21].

## Methods

We purchased short-term radon test kits from Enviro Sciences/Alpha Energy, Inc. (Carrollton, TX) that were specially bar-coded for this study. The kits included a pre-addressed, pre-paid envelope for returning the kit to the company for testing. The bar-coding enabled us to determine the number of kits ordered by participants and whether they had been actually used (i.e., returned to the laboratory for analysis).

### Sample

Study participants were recruited from an undergraduate-level introductory communication class at the University of North Dakota. Residential radon levels in North Dakota are among the highest in the U.S. [5]. Participants installed the radon app on their smartphones and used it for a month. We used a pretest-posttest design to test the feasibility and effectiveness of the app. Participants received extra credit and a gift card in exchange for their participation in the baseline, pre-exposure test (T1) and received additional extra credit, a second gift card, and a radon-themed University t-shirt in exchange for participation in the post-exposure test (T2). Participants were given access to a computer and completed an online informed consent and questionnaire in both T1 and T2. This research was approved by the IRB at the University of North Dakota, which considered the study exempt. All students read a standard consent form.

### Participants’ characteristics

Table 1 presents participants’ demographic characteristics. The average age was 21 (SD = 2.0). Females (*n* = 58 [60.4%] in T1; *n* = 55 [59.1%] in T2) slightly outnumbered males. Most were non-Hispanic White (*n* = 84 [87.5%] in T1; *n* = 80 [86.0%] in T2). The annual household income of approximately 40% of participants was $100,000 or higher (*n* = 32 [41.6%] in T1; *n* = 28 [38.4%] in T2). Over 80% of participants reported that they were non-smokers (*n* = 79 [82.3%] in T1).

**Table 1:** Demographic characteristics in pre-exposure and post-exposure tests

Characteristics	T1 (*N* = 97)	T2 (*N* = 93)	Test statistics	*p* value
**Age, mean (SD)**	21.0 (2.0)		–	–
**Sex**				
Male	38 (39.6)	38 (39.2)	.00	1.00^a^
Female	58 (60.4)	55 (59.1)		
**Race/ethnicity**				
Non-Hispanic White	84 (87.5)	80 (86.0)	.00	1.00^a^
Non-White or Hispanic	12 (12.5)	13 (14.0)		
**Annual household income**				
Lower than $100,000	45 (58.4)	45 (61.6)	.80	.38^a^
$100,000 or higher	32 (41.6)	28 (38.4)		
**Smoking status**				
Non-smokers	79 (82.3)	76 (81.7)	.80	.38^a^
Smokers	17 (17.7)	17 (18.3)		

*Note*: T1, pre-exposure test; T2, post-exposure test; −, not applicable; *SD* standard deviation

^a^calculated by related-samples McNemar Change test

### Measurement

Two *behavioral outcomes* were recorded based on participants’ actual behaviors: requesting a free radon test and sending the kit to the testing lab. Similar to the process of returning an at-home disease screening test, we considered sending the test kit to the lab to represent “*test process completion*”. We measured *radon knowledge* as the percentage of accurate responses to 20 True-False statements (e.g., “The EPA has determined that homes with 4 picocuries per liter [pCi/L] or more of radon should be fixed”). Two radon knowledge index variables were created by summing participants’ responses to each statement in T1 and T2. *Attitudes toward radon testing* were measured by five 7-point semantic differential scales [18]. Each of the four variables, *perceived severity of the threat*, *perceived susceptibility of the threat*, *response efficacy*, and *self-efficacy* was measured by two 7-point Likert scales [21, 22]. All of the measurement items used in this study were adopted from statements in EPA brochures (e.g., [2]) and previous studies that found acceptable reliability and validity [18, 21, 22]. Table 2 presents measurement scales and reliability statistics in T1 and T2. We compared differences in responses in T1 and T2 using a two-tailed paired-sample *t*-test. *P-*values ≤  0.05 were considered significant.

**Table 2:** Measurement Scales and Reliability Statistics

Measurement scales	T1	T2
*Attitudes toward radon testing*:		
bad – good, negative – positive, unfavorable – favorable, dislike – like, and worthless – valuable	.95^a^	.95^a^
*Perceived severity of the threat:*		
• I feel lung cancer would be a very serious illness for me. • If I had lung cancer, my whole life would change.	.62^b^	.70^b^
*Perceived susceptibility of the threat:*		
• I am more likely to get lung cancer because of how I live my life. • Personally, I feel vulnerable to developing lung cancer.	.74^b^	.86^b^
*Response efficacy*:		
• I feel that a radon test would help me personally to reduce my risk of lung cancer. • I have a lot to gain from conducting a radon test in my home.	.80^b^	.91^b^
*Self-efficacy*:		
• If I wanted, I could easily perform a radon test. • I feel like I would know how to test my home for radon if I wanted to.	.79^b^	.81^b^

^a^ calculated by Cronbach’s alpha

^b^ calculated by Spearman-Brown coefficient

## Results

### Effects of the radon app on knowledge, testing attitudes, threat and coping appraisals, and behaviors

A total of 97 participants participated in T1, and 93 participants (96%) followed up in T2. Table 3 presents paired-samples *t*-test results comparing radon knowledge, attitudes, and perceptions between T1 and T2. After using the radon app for a month, participants’ level of radon knowledge increased significantly (*p* < .001). In particular, participants showed a significantly higher accuracy in responses in terms of the importance of testing houses for radon (T1 = 46.74%; T2 = 83.70%; 95% Confidence Interval [CI] in difference = 25.88, 46.74; McNemar χ^2^ = 32.11; *p* < .001); radon exposure as a risk factor for lung cancer (T1 = 52.17%; T2 = 81.52%; 95% CI in difference = 18.07, 39.61; McNemar χ^2^ = 17.79; *p* < .001), and home remediation as a way to address higher than four pCi/L radon levels (T1 = 32.61%; T2 = 66.39%; 95% CI in difference = 22.91, 45.01; McNemar χ^2^ = 26.95; *p* < .001).

**Table 3:** Paired-samples *t*-test results comparing radon knowledge, attitudes, and threat and coping appraisal variables between pre-exposure and post-exposure tests

Variables	Pre-exposure test (*N* = 97) Mean (SD)	Post-exposure test (*N* = 93) Mean (SD)	*t (df*) ^a^	*p* value
Radon knowledge	6.45 (4.77)	10.42 (4.59)	7.69 (91)	< .001
Attitudes toward radon testing	5.40 (1.34)	6.31 (.91)	6.08 (91)	< .001
Perceived severity	6.60 (.92)	6.64 (.82)	.45 (91)	.66
Perceived susceptibility	3.03 (1.65)	3.26 (1.73)	1.22 (91)	.23
Response efficacy	4.93 (1.37)	5.38 (1.06)	3.01 (91)	<.01
Self-efficacy	3.60 (1.26)	5.29 (1.10)	11.83 (91)	<.001

^a^ calculated by paired-samples *t*-test

Attitudes toward radon testing became more positive in T2 than in T1 (*p* < .001). Conversely, threat appraisal – perceived severity (*p* > .05) and perceived susceptibility (*p* > .05) – did not differ significantly between T1 and T2. In contrast, participants’ response efficacy was significantly higher in T2 than T1 (*p* < .001). In other words, the use of the radon app helped participants to believe that testing their house for radon level is an effective way to prevent lung cancer. Moreover, there was a significant increase in participants’ self-efficacy in T2 (*p* < .001). This indicates that use of the radon app enabled participants to believe that they can actually test their home.

Most importantly, the radon app effectively initiated the desired  behavioral responses. Twenty-three [23] participants (24%) utilized the radon app to request a free radon test kit. The test process completion rate was 9%; that is, among 23 participants who ordered the test kit, two used the kits to test their house and sent the kit to the lab for analysis.

### Predictors of ordering a free radon test kit

We used logistic regression to examine whether radon knowledge, attitudes toward radon testing, perceived severity, perceived susceptibility, response efficacy, and self-efficacy predicted ordering a free radon test kit, controlling for gender, age, race/ethnicity, household income, and current smoking status. The results of the logistic regression are shown in Table 4. Gender, race/ethnicity, household income, and current smoking status were not associated with ordering a test kit. Age was a positive predictor of ordering the test kit in the first block (B = .32, SE = .13, Wald = 6.22, Exp(B) = 1.37, 95% CI = 1.07, 1.76, *p* < .05). When all predictors were included in the second block, age was no longer significant. Radon knowledge, attitudes toward radon testing, perceived severity, and perceived susceptibility were not significant predictors. Conversely, response efficacy (B = 1.67, SE = .52, Wald = 10.38, Exp(B) = 5.34, 95% CI = 1.93, 14.78, *p* < .01) and self-efficacy (B = 1.38, SE = .56, Wald = 6.08, Exp(B) = 3.97, 95% CI = 1.33, 11.90, *p* < .05) were significant positive predictors of ordering the test kit. In other words, individuals who viewed radon testing as a way to cope with the radon threat and believed in their ability to perform the test were more likely to order a test kit.

**Table 4:** Logistic regression result predicting ordering a free radon test kit

	B	SE	Wald	Exp(B)	95% CI	*p* value
Block 1						
Age	.32	.13	6.22	1.37	1.07, 1.76	< .05
	Chi-square = 7.12, *df* = 1, *p* < .01; −2 log likelihood = 94.10; Cox & Snell *R*^2^ = .07; Nagelkerke *R*^2^ = .11					
Block 2						
Age	.30	.18	2.72	1.35	.95, 1.93	.10
Response efficacy	1.67	.52	10.38	5.34	1.93, 14.78	< .01
Self-efficacy	1.38	.56	6.08	3.97	1.33, 11.90	< .05
	Chi-square = 49.21, *df* = 3, *p* < .001; −2 log likelihood = 52.00; Cox & Snell *R*^2^ = .41; Nagelkerke *R*^2^ = .62					

## Discussion

### Summary of findings and limitations

We designed a radon education app as a communication tool to increase radon awareness. Our most important findings were the positive effects of radon app on knowledge, attitudes toward testing, and coping appraisal. We found that the concepts of self-efficacy and response efficacy were significant predictors of ordering a radon test kit.

The app was clearly effective in increasing radon knowledge. Participants were able to correctly identify the threat (i.e., radon gas), the consequence of the threat (i.e., lung cancer), and how to prevent it (i.e., via testing the house and utilizing a radon mitigation system). These are the core elements of radon risk communication. The positive effects of app on improved knowledge suggest that delivering content in short, simple terms via a mobile app is an effective way to communicate radon-related information. The app is a very different style of content delivery than the explanations typically used in pamphlets and brochures [11, 12] or in telephone interviews [9, 10]. With several notable exceptions [8, 12, 23] – e.g., an increase in radon test kit purchases reported among individuals exposed to digital signs in physicians’ waiting rooms [8] – few intervention studies have reported that the intervention improved individuals’ knowledge, attitudes, perceptions, or behaviors.

The levels of radon knowledge and attitudes toward radon testing increased in the post-exposure test, a finding consistent with the results of several studies regarding radon knowledge [12]. Notably, improved knowledge and attitudes by themselves were not significant predictors of ordering the test kit. Many studies have assumed that the critical problem underlying low radon testing and home remediation rates is individuals’ lack of understanding about radon. On this view, the primary objective of radon intervention strategies has been information delivery [24]. This assumption is based on the deficit model [25] whereby individuals fail to perform positive behaviors due to the lack of knowledge or understanding. Conversely, our findings indicate that an increase in individuals’ knowledge does not reliably predict positive behavioral changes.

We found that coping appraisals – response efficacy and self-efficacy – were the most significant predictors of ordering a test kit, which is consistent with previous studies [23]. Interestingly, threat appraisals – perceived severity and perceived susceptibility – did not increase the likelihood of ordering a test kit. This could be because participants already understood the severity of the threat even before using the app in T1. Despite a high level of perceived severity, perceived susceptibility was consistently low both in T1 and T2. This is consistent with the findings of many studies of radon knowledge, which demonstrate that respondents display “optimistic bias”, i.e., the view that radon is more of a danger to *others* than to themselves [7]. Additionally, young individuals in particular often do not consider themselves to be at risk of cancer or death [26].

Our study has several limitations. First, use of a student sample limits the generalizability of our findings. Thus, these findings should be replicated with a more generalizable sample. Because our primary focus was testing the app’s feasibility, we did not perform power analyses a priori in order to determine the optimum sample size. Additionally, the ultimate target audience of a radon-education app is homeowners/homebuyers. Compared to undergraduates, homeowners tend to be older, e.g., the median age of first time home-buyers in the US is 33 [27] and older individuals may have lower smartphone literacy [28]. Secondly, our pretest-posttest design did not include an unexposed group. Finally, some idealized conditions applied, i.e., the app was installed on participants’ phones. In the “real world,” there may be disincentives to install such software, which may be regarded as undesirable (i.e., “bloatware”).

Conversely, our study has several strengths. To our knowledge, this is the first study to use a smartphone app to address radon knowledge and beliefs. The app significantly improved radon knowledge and coping appraisals. Notably, our use of coded test kits allowed us to measure actual ordering and testing behaviors as well as view the lab results for completed radon tests. Nearly a quarter of participants (24%) used the app to order the free test kit. However, of the participants who ordered the free test kit, only two ultimately returned the kit to the laboratory, resulting in a test process completion rate of 9%. This relatively low rate is consistent with the interpretation of Doyleand colleagues [13] who emphasized the many opportunities for “failure” of educational campaigns to produce home mitigation; e.g., individuals first must learn about radon, obtain a test kit, use the test kit, return the kit, interpret the test results, and, if warranted, ultimately remediate their home, a process which itself contains numerous opportunities for lack of follow through. Although our results did not involve individuals who remediated their homes, our findings identified the step, “using the test kit” as an important opportunity for failure in the overall goal of home remediation. This vulnerability is faced by many public health interventions, e.g., “at home” disease screening tests which often are not returned to the laboratory after they are obtained [29]. However, a smartphone app is ideally suited to the use of methods to increase test kit use, such as the addition of in-app reminders to individuals who have received the test kits but not yet returned them. The use of such reminders would be a logical and feasible next step to improve the test process completion rate.

## Conclusions

Residential radon is an important cause of preventable mortality, but it has proven very difficult to motivate individuals to take action against an invisible threat, like radon, whose health consequences appear decades after chronic exposure [7]. Our response to this challenge was to create a radon app for smartphones. Individuals’ exposure to app content significantly improved their radon knowledge, attitudes toward radon testing, response efficacy, and self-efficacy. In addition, the app motivated nearly a quarter of participants to order a free radon test kit. We conclude that a radon app for smartphones is a promising tool for improving radon knowledge and testing behavior.

## Data Availability

The data sets generated and/or analysed in the current study are not publicly available but the data are available from the corresponding author on reasonable request.

## Abbreviations

App: Smartphone application (software)
CI: Confidence Interval
EPA: U.S. Environmental Protection Agency
IRB: Institutional Review Board
NC: North Carolina
ND: North Dakota
SD: Standard Deviation
T1: Pre-exposure Test
T2: Post-exposure Test
TX: Texas
